# Crucial Insights from Interictal 99mTc–ECD Brain Perfusion SPECT in Enhancing Pediatric Epilepsy Diagnosis and Management

**DOI:** 10.1055/s-0045-1812326

**Published:** 2025-11-12

**Authors:** Gupta Sarath Chandra Sanisetty, Manishi L. Narayan, Prateek Kumar Panda, Indar Kumar Sharawat, Ramya S.

**Affiliations:** 1 Department of Nuclear Medicine, All India Institute of Medical Sciences, Rishikesh, Rishikesh, Uttarakhand, India; 2 Department of Pediatrics, All India Institute of Medical Sciences, Rishikesh, Rishikesh, Uttarakhand, India

**Keywords:** seizure onset zone, temporal lobe epilepsy, 99mTc-ECD brain perfusion SPECT, drug-resistant epilepsy, interictal-ictal SPECT

## Abstract

**Objective:**

Epilepsy, a prevalent neurological disorder in children, manifests through recurrent seizures due to abnormal neuronal activity. Drug-resistant epilepsy (DRE) is a significant clinical challenge, often necessitating advanced diagnostic approaches to optimize treatment strategies. Single photon emission computed tomography (SPECT) imaging, particularly using the radiopharmaceutical 99mTc-ethyl cysteinate dimer (99mTc-ECD), offers potential in localizing epileptogenic zones, especially when conventional magnetic resonance imaging (MRI) and electroencephalography (EEG) findings are inconclusive. This study was performed to evaluate patterns of brain perfusion using 99mTc-ECD SPECT in children aged < 18 years diagnosed with DRE, and to correlate these findings with clinical evaluations, MRI, and video EEG reports.

**Materials and Methods:**

A descriptive observational study was conducted over 18 months on 60 children with DRE. Each participant underwent a detailed clinical history, video EEG, MRI, and a 99mTc-ECD brain perfusion SPECT scan. The SPECT images were analyzed visually and semiquantitatively for hypoperfusion and hyperperfusion patterns. Data were cross-referenced with clinical lateralization, MRI findings, and EEG results to assess concordance.

**Results:**

A total of 60 children with DRE and mean age of 11.02 + 3.8 years (1–< 18 years) including 16 (26.7%) female and 44 (73.3%) male patients were prospectively analyzed. Forty of 60 (66.7%) patients had normal MRI brain study and 23/60 (38.3%) patients had normal EEG records. Medial temporal lobe hypoperfusion was seen in all patients, while 16/60 (26.67%) patients had additional foci of hyperperfusion in left frontal lobe and 14/60 (23.3%) patients in right frontal lobe. Thirty-six of 60 (60%) patients had involvement of one hemisphere, 24/60 (40%) showed perfusion abnormalities in both hemispheres. Moderate to severe hypoperfusion was seen in left temporal lobe in 39/60 (65%) patients whereas 30/60 (50%) patients showed moderate to severe hypoperfusion in right temporal lobe. SPECT findings were concordant with EEG in 16/60 (26.67%) of cases and concordant MRI, EEG, and 99mTc-ECD was seen in 5/60 (8.3%) cases.

**Conclusion:**

99mTc-ECD brain perfusion SPECT is a valuable diagnostic tool in the comprehensive evaluation of pediatric DRE. It enhances lateralization and localization of epileptogenic zones, especially when conventional imaging is nondiagnostic. Interictal brain SPECT, is easily available, indispensable, and complementary in diagnosis. Combining it with clinical and electrophysiological evaluation can significantly improve clinical outcome and management of patients with DRE.

## Introduction

Epilepsy is a brain disorder characterized by recurrent seizures and it reflects underlying brain dysfunction that is variable and multifactorial. Seizures are characterized by abnormal excessive synchronous neuronal activity in the brain. 1

There are 70 million persons with epilepsy worldwide and the prevalence of epilepsy across the globe is estimated to be 5 to 9/1,000 population. There are nearly 12 million persons with epilepsy expected to reside in India. They contribute to nearly one-sixth of the entire global burden. 2 3

Epilepsy is also one of the most common neurological disorders in children with reasonably favorable long-term outcomes with nearly two-thirds achieving seizure freedom. But, one-third of children with early pharmacological resistance finally achieve seizure control. The prognosis is worse for patients with abnormal neuroimaging and may require early surgical intervention. Interictal and ictal single photon emission computed tomography (SPECT) in pediatric population help in the identification of epileptogenic focus that can help in presurgical planning and placement of intracranial electrodes, thereby improving the outcome in this subset of patients with medically intractable/drug-resistant epilepsy (DRE). 4

Video electroencephalography (EEG) is the modality of choice for the localization of seizures initially. EEG being a noninvasive technique is done as an initial investigation. Rapidly spreading seizures and deep lesions cannot be accurately identified by scalp EEG. Magnetic resonance imaging (MRI) is the modality of choice for the identification of morphologic and functional abnormalities to complement the video EEG information. Lesional epilepsy of the temporal lobe, including mesial temporal sclerosis, dysplasia, migration disorders, tumors, and vascular malformations, can be identified with MRI. Functional imaging plays an important role in the care of patients with nonlesional epilepsy. 5

Cerebral perfusion and metabolism are different processes but are coupled in most physiologic and pathologic conditions. Molecular imaging of brain perfusion with SPECT is an established functional imaging modality for measuring regional cerebral blood flow in vivo. Regional brain glucose metabolism is shown by 18-fluorodeoxyglucose (18F-FDG) positron emission tomography (PET). 1 5

Cerebral blood flow is known to change according to the seizure course within the epileptogenic zone, that is, the ictal onset area. Between seizures, cerebral blood flow is decreased (“interictal hypoperfusion”), whereas it is increased during seizures (“ictal hyperperfusion”). Cerebral blood flow deeply breaks down immediately after the end of the seizure (“post-ictal hypoperfusion”) and remains at this very low level for several minutes before it goes back to its interictal level. 1 6

SPECT and PET imaging techniques are aimed at the localization or lateralization of the epileptogenic cortex. They are useful for the subsequent subdural placement of electrodes, especially when MRI results are normal. They reduce the number of invasive EEGs and show focal-abnormal metabolic regions. SPECT and PET play an essential role in the reclassification of the functional status of the brain and define the functional integrity of the brain, give additional information on surgical opportunities for patients, and understand the pathophysiology of epilepsy. 1 The study was conducted to emphasize the important diagnostic role of 99mTc-ethyl cysteinate dimer (ECD) in localizing epileptogenic cortex.

## Material and Methods

This was a descriptive observational study conducted 18 months from the date of approval from institutional ethics board approval (AIIMS/IEC/22/649, dated 23.12.2022). The study included 60 children with diagnosis of DRE aged 1 to 18 years after being thoroughly evaluated by pediatric neurologist according to the International League Against Epilepsy criteria 7 8 who had focal/generalized epilepsy based on clinical/radiological/EEG findings. 9 10 11 The study excluded patients who had presence of chronic major systemic illness making patient unfit for the scan and patients with self-limited focal/generalized epilepsy syndromes.

All the patients were injected 0.2 to 0.3 mCi/kg (7.4–11.1 MBq), a minimum dose of 3 to 5 mCi (111–185 MBq), of 99mTc-ECD intravenously as a slow bolus over approximately 20 seconds followed by saline flush in resting position. Tracer was injected intravenously in a quiet room with dim light with the patient seated or reclined comfortably and avoided interaction, before, during, or for 5 minutes after injection to avoid any sensory and cognitive stimulation, which may affect brain perfusion. For patients who were uncooperative (those with severe cognitive impairment or with loss of insight) and require sedation, tracer injection was given prior to sedation to avoid sedation-induced blood flow changes. The tracer was administered following meticulous radiolabeling and routine quality control checks. SPECT images of the brain were acquired approximately 1 hour after injection using a dual-head camera, GE-NMCT 670, SPECT-computed tomography (CT) (GE Healthcare). 12 13

99mTc-ECD brain SPECT Images were analyzed by an experienced nuclear medicine physician. Biodistribution image was acquired 15 to 30 minutes after administration of radiotracer and were evaluated for efficacy of radiolabelling. SPECT images were reconstructed in axial, coronal, and sagittal planes. Images were critically examined during interpretation for the presence of head motion, attenuation artifacts, and other artifacts arising from gamma camera quality control. Image reconstruction performed using filtered back projection technique and Butterworth filter with critical frequency was set at 0.45. After reconstruction images were attenuation corrected using Chang's attenuation correction method. Perfusion defects were categorized as mild, moderate, and severe, considering normal cerebellar uptake as reference standard (except in two cases where cerebellar uptake was abnormal). Additionally, semiquantitative analysis of images was performed using three-dimensional stereotactic surface projection image and *Z* -scores were calculated using Q-brain 4.0117 software (GE Healthcare, 2016). SPECT imaging findings were correlated with clinical evaluations, MRI, and video EEG reports.

### Statistical Analysis

1. All the data was entered in an Excel spreadsheet and was analyzed using SPSS version 26.0 for Windows OS.
2. Categorical variables were presented using frequency and 95% confidence interval, and continuous variables were mentioned in mean/standard deviation or median/interquartile range.
3. Difference in distribution of categorical variables between two groups was tested for statistical significance using the Fisher's exact test/chi-square test.
4. A *p* -value of < 0.05 is considered statistically significant.

## Results

A total of 60 patients with seizure disorder and clinical diagnosis of DRE (female, *n* = 16 [26.7%]; male, *n* = 44 [73.3%]) ( Table 1 ) referred for 99mTc-ECD brain perfusion SPECT after thorough examination by a pediatric neurologist with fulfilling the eligibility criteria were evaluated in this study. The mean age was 11.02 ± 3.88 years (range = 1–18 years).

**Table 1:** Distribution of participants by age group and sex

Age groups	Frequency	Percent (%)
1–5 years	7	11.7
5–10 years	14	23.3
10–15 years	33	55.0
15–20 years	6	10.0
Sex		
Female	16	26.7
Male	44	73.3
Total	60	100.0

The data indicated that the majority of patients *n* = 41/60 (68.3%) had seizures for more than 1 year. In contrast, *n* = 19/60 (31.7%) of the patients had seizures for more than 3 months and less than 1 year. The study revealed that the participants have a wide array of clinical presentation and diagnoses, with the most common presentation being drug-resistant right-sided focal seizures and left-sided focal seizures, affecting *n* = 18 (30%) and *n* = 8 (13.3%) of the participants, respectively. Other frequently reported conditions included drug-resistant temporal lobe epilepsy (TLE) (5%) and mesial temporal lobe sclerosis (5%). Numerous other presentations include generalized tonic-clonic seizures (GTCSs), dystonia, sleep onset epilepsy, hypermotor epilepsy, juvenile myoclonic epilepsy, and structural brain abnormalities, each reported in 1.7% of the cases. The current DRE cohort (1–18 years age) shows diverse clinical presentations in the population of Uttarakhand region of North India. The most common clinical presentation was right-sided focal seizures (30%) followed by left-sided focal seizures (13.3%). Numerous other presentations include GTCS, dystonia, sleep onset epilepsy, hypermotor epilepsy, juvenile myoclonic epilepsy, and structural epilepsy (1.7% each).

The lateralization of the epileptogenic focus using clinical assessments, MRI, EEG, and 99mTc-ECD brain perfusion SPECT was analyzed. Clinically, 25 (41.7%) cases were right-lateralized, 11 (18.3%) were left-lateralized, and 24 (40%) had no lateralization. MRI results showed that 40 (66.7%) participants had no lateralization, while 8 (13.3%) were right-lateralized and 7 (11.7%) were left-lateralized. EEG findings indicated that 16 (26.7%) were right-lateralized, 14 (23.3%) were left-lateralized, and 23 (38.3%) showed no lateralization. ECD results revealed 24 (40%) bilateral, 21 (35.0%) left-lateralized, and 15 (25%) right-lateralized cases. These results highlighted variability in lateralization detection across different diagnostic tools.

The distribution of lateralization patterns among different diagnostic modalities, including MRI scans, EEG recordings, and 99mTc-ECD studies, was analyzed ( Table 2 ).

**Table 2:** Distribution of lateralization patterns among different diagnostic modalities, including MRI scans, EEG recordings, and 99mTc-ECD studies

Lateralization	Categories	Total ( *n* = 60)	Chi-square value, df	*p* -value a
MRI	N	40	2.271, 3	0.518
		66.70%		
	B/L	5		
		8.30%		
	L	7		
		11.70%		
	R	8		
		13.30%		
EEG	N	23	2.323, 3	0.508
		38.30%		
	B/L	7		
		11.70%		
	L	14		
		23.30%		
	R	16		
		26.70%		
ECD	B/L	24	1.918, 2	0.383
		40.00%		
	L	21		
		35.00%		
	R	15		
		25.00%		

Abbreviations: B/L, bilateral; df, degree of freedom; ECD, ethyl cysteinate dimer; EEG, electroencephalography; L, left; MRI, magnetic resonance imaging; N, no lateralization; R, right.

a Chi-square test, *p* < 0.05 (significant).

Globally, no statistically significant difference in the lateralization patterns of the epileptogenic zone is seen when comparing the results from MRI, EEG, and 99mTc-ECD SPECT modalities. The *p* -values for MRI (0.518), EEG (0.508), and ECD (0.383) exceed the typical alpha level of 0.05, suggesting that the observed distributions are likely due to chance (MRI: chi-square = 2.271, *p* = 0.518; EEG: chi-square = 2.323, *p* = 0.508; ECD: chi-square = 1.918, *p* = 0.383).

Out of 60 patients undergoing 99mTc-ECD brain perfusion SPECT, 6 patients (10%) had frontal hypoperfusion, 10 patients (16.7%) had parietal hypoperfusion, majority of patients had temporal hypoperfusion, but none of the patients had predominant occipital hypoperfusion. Summary of perfusion defects with respect to region, localization, laterality, and severity is shown in Table 3 .

**Table 3:** Summary of perfusion defects with respect to region localization, laterality, and severity on 99mTc-ECD interictal SPECT

Region	Severity of hypoperfusion	No. of patients	Severity of hyperperfusion	No. of patients
LF	Mild	11 [18.3%]	Mild	16 (26.7%)
	Moderate	5 [8.3%]	−	−
	Severe	1 [1.7%]	−	−
	N	43 [71.7%]	N	44 (73.3%)
RF	Mild	10 [16.7%]	Mild	14 (23.3%)
	Moderate	7 [11.7%]	−	−
	Severe	2 [3.3%]	−	−
	N	41 [68.3%]	N	46 (76.7%)
LP	Mild	3 [5.0%]	Mild	8 (13.3%)
	Moderate	3 [5.0%]	−	−
	Severe	1 (1.7%)	−	−
	N	54 [90.0%]	N	51 (85.0%)
RP	Mild	1 [1.7%]	Mild	8 (13.3%)
	Moderate	1 [1.7%]	−	−
	Severe	3 [5.0%]	−	−
	N	55 [91.7%]	N	52 (86.7%)
LT	Mild	18 [30.0%]	Mild	1 (1.7%)
	Moderate	35 [58.3%]	−	−
	Severe	4 [6.7%]	−	−
	N	3 [5.0%]	N	59 (98.3%)
RT	Mild	16 [26.7%]	Mild	1 (1.7%)
	Moderate	25 [41.7%]	−	−
	Severe	5 [8.3%]	−	−
	N	14 [23.3%]	N	59 (98.3%)
LO	Mild	2 [3.3%]	Mild	1 (1.7%)
	Moderate	2 [3.3%]	−	−
	N	56 [93.3%]	N	59 (98.3%)
RO	Mild	2 [3.3%]	Mild	1 (1.7%)
	Moderate	2 [3.3%]	−	−
	N	56 [93.3%]	−	−
LBG	N	53 [88.3%]	N	60 (100.0%)
	Mild	7 [11.7%]	−	−
RBG	Mild	11 [18.3%]	−	−
	N	49 [81.7%]	N	60 (100.0%)
LTh	Mild	1 [1.7%]	−	−
	N	59 [98.3%]	N	60 (100.0%)
RTh	Mild	1 [1.7%]	−	−
	N	59 [98.3%]	N	60 (100.0%)
LCb	Mild	−	Mild	1 (1.7%)
	N	60 [100.0%]	N	59 (98.3%)
RCb	Mild	1 [1.7%]	Mild	1 (1.7%)
	N	59 [98.3%]	N	59 (98.3%)

Abbreviations: ECD, ethyl cysteinate dimer; LBG, left basal ganglia; LCb, left cerebellum; LF, left frontal; LO, left occipital; LP, left parietal; LT, left temporal; LTh, left thalamus; N, normal; RBG, right basal ganglia; RCb, right cerebellum; RF, right frontal; RO, right occipital; RP, right parietal; RT, right temporal; RTh, right thalamus; SPECT, single photon emission computed tomography.

Semiquantitative analysis of 99mTc-ECD uptake of temporal lobe was performed for all the patients ( *n* = 60*). The ratio of 99mTc-ECD uptake between cerebellum to the medial temporal lobe was estimated. Cerebellar uptake is considered as reference standard in this analysis. The average ratio of cerebellum/right medial temporal lobe was 0.87 and the average ratio of cerebellum to left medial temporal lobe was 0.86 ( Table 4 ). This showed that the majority of patients (58/60) had medial temporal lobe hypoperfusion.

**Table 4:** Semiquantitative analysis of 99mTc-ECD uptake with cerebellum/medial temporal lobe ratios of all the patients ( *n* = 60)

Cerebellum/Medial temporal lobe		
Case	Right	Left
1	0.81	0.86
2	0.9	0.91
3	0.86	0.87
4	0.89	0.86
5	1.07	0.95
6	0.89	0.89
7	0.84	0.9
8	0.8	0.8
9	0.97	0.98
10	0.8	0.82
11	0.87	0.9
12	0.7	0.7
13	1.02	0.7
14	0.85	0.79
15	0.8	0.8
16	0.9	0.9
17	0.95	0.92
18	0.8	0.88
19	0.91	0.89
20	0.9	0.89
21	0.86	0.92
22	0.86	0.75
23	0.73	0.81
24	0.89	0.82
25	0.95	0.86
26	0.92	0.91
27	0.82	0.86
28	0.89	0.88
29	0.89	0.94
30	1.02	0.77
31 a	0.79	0.81
32	0.86	0.82
33	0.87	0.83
34	0.88	0.83
35	0.87	0.87
36	0.91	0.93
37	0.87	0.87
38	0.85	0.91
39	0.85	0.81
40	0.85	0.83
41	0.75	0.84
42	0.75	0.74
43	0.85	0.89
44	0.8	0.84
45	0.73	0.81
46	0.94	0.94
47	0.93	0.99
48	0.9	0.87
49	0.8	0.88
50	0.89	0.86
51	1.02	0.77
52	0.92	0.9
53	0.9	0.86
54	0.93	0.91
55 a	0.92	0.86
56	0.8	0.84
57	0.91	0.89
58	1.06	1.04
59	0.9	0.9
60	0.93	0.86

Abbreviation: ECD, ethyl cysteinate dimer.

a Excluded from analysis.

Case #31 KNI – bilateral cerebellar hyperperfusion.

Case #55 JS – Right cerebellar hypoperfusion.

This study analyzed the clinical outcomes and management changes in patients. Follow-up clinical control showed that 45 (75%) patients were seizure free after 12 months of follow-up, while 15 (25%) did not. Out of these 15 patients, 8/15 (53.3%) were noncompliant on medication. Five of 15 (33.3%) were lost to follow-up. One of 15 (6.67%) who had craniopharyngioma did not undergo surgery. One patient expired on follow-up. These findings suggest that a majority of patients who experienced positive outcome did not require changes in their management plans. The study also indicated that nearly all patients (98.3%) did not require management changes to achieve clinical control, with consistent results across all age groups. In the 10 to 15 age groups, one patient expired, representing the only case of mortality in the study, which was 1.7% of the total cases. No patients in the other age groups expired.

## Discussion

The study consisted of 60 participants, with the majority (55%) being between 10 and 15 years of age. Males accounted for 73.3% of the participants. The age group of 15 to 20 years had the lowest representation, making up only 10% of the participants. This implies more males were involved in the study and the participants were predominantly in 10 to 15 years of age ( Table 1 ). The age and gender distribution in this study is similar to what other epilepsy studies have shown. A systematic review by Fiest et al stated that children and the elderly population are the most affected by epilepsy. 6 The male dominance here is also in tune with some prior research studies.

In the current study, participant demographics combined with their individual diagnoses reveal that drug-resistant right- and left-sided focal seizures were the most common presentation. There were other presentations such as GTCSs, dystonia, sleep onset epilepsy, hypermotor epilepsy, juvenile myoclonic epilepsy, and structural brain abnormalities, each reported in 1.7% of the cases. The distribution showcases the multifactorial nature of epilepsy and seizure disorders throughout the population. This broad range of epilepsy diagnoses seen in our study is indicative of the heterogeneity of the disorder as described in the literature. 14

This research reports a broader range of specific diagnoses, possibly due to differences in diagnostic categories or characteristics of the population. The high prevalence of TLE as a focal epilepsy syndrome is evidenced by the comparatively large number of cases seen in this context. A total of 31/60 (51.7%) patients received a prescription for combination of two antiepileptic medications, in 13% patients three antiepileptic drugs (AEDs) were used, while 10% cases all four AEDs were administered ( Table 5 ). These findings concur with those from previous research on refractory epilepsy, which found that patients with DREs were prescribed multiple medications at a higher rate than their peers in the general population.

**Table 5:** Distribution of patients by number of antiepileptic drugs (AEDs) used

No. of AED	Frequency	Percent (%)
2	31	51.7
3	19	31.7
4	6	10.0
5	1	1.7
6	3	5.0
Total	60	100.0

Chen et al, in a 30-year long cohort study published in 2019, emphasized that despite availability of many new AEDs with differing mechanism of action, overall outcome in newly diagnosed epilepsy has not been improved. Most patients who attain optimal seizure control do so with the first of second AED. The probability of attaining seizure freedom diminishes with subsequent regimen tried. 14

**Fig. 1:**
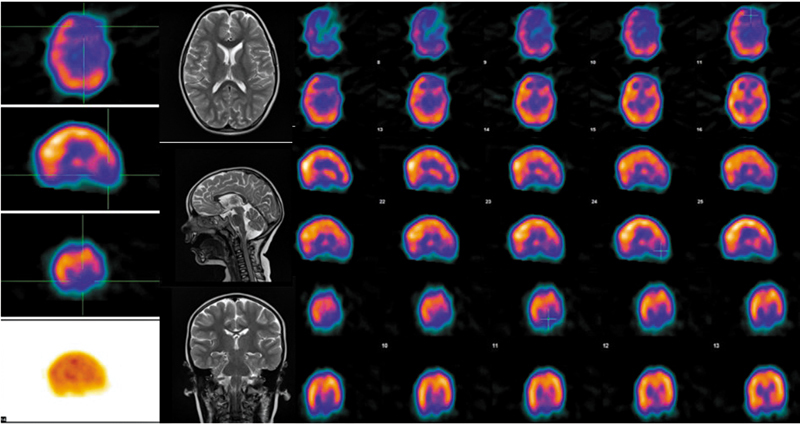
Interictal 99mTc-ethyl cysteinate dimer (ECD) brain perfusion single photon emission computed tomography (SPECT) images in a 1-year-old female patient with recurrent right focal seizures. Transaxial, sagittal, and coronal sections showing moderate to severe hypoperfusion in left frontal and temporal cortex. Magnetic resonance imaging (MRI) of brain was unremarkable.

Visual assessment of the medial temporal region is difficult because of its inherently low ECD uptake, our patient population also showed significantly reduced uptake in this region on visual as well as semiquantitative analysis ( Table 4 ). Other investigators 15 16 17 have also found significant decrease in ECD uptake of temporal cortex on visual analysis. They suggested low uptake in the hippocampus might result in false negative visual interpretation in patients with dementia or epilepsy. 16 17 Therefore, quantitative analysis of hippocampus would be better suited for the evaluation of hippocampal abnormalities on 99mTc-ECD images. 16 17 Our study showed that in patients where EEG and MRI were unremarkable (16/60), 99mTc-ECD SPECT helped in laterality as well as severity of perfusion defect. In these cases, 99mTc-ECD perfusion SPECT has proven to be invaluable ( Table 6 and Fig. 1 ). The findings of EEG and 99mTc-ECD were concordant in 16/60 (26.67%) patients where MRI was unremarkable. MRI and 99mTc-ECD scan findings were concordant in 2/60 (3.4%) patients where EEG was unremarkable. Findings of all three modalities, EEG, MRI, and 99mTc-ECD, were concordant in 5/60 (8.3%) patients.

**Table 6:** EEG and MRI unremarkable but 99mTc-ECD showing perfusion defects ( *n* = 16)

	99mTc-ECD	MRI	EEG
Case 3	B/L	N	N
Case 11	B/L	N	N
Case 14	B/L	N	N
Case 17	L	N	N
Case 23	B/L	N	N
Case 25	L	N	N
Case 26	R	N	N
Case 28	B/L	N	N
Case 30	L	N	N
Case 31	B/L	N	N
Case 33	L	N	N
Case 40	L	N	N
Case 42	R	N	N
Case 43	L	N	N
Case 44	R	N	N
Case 56	R	N	N

Abbreviations: B/L, bilateral; ECD, ethyl cysteinate dimer; EEG, electroencephalography; L, left; MRI, magnetic resonance imaging; N, normal; R, right.

**Fig. 2:**
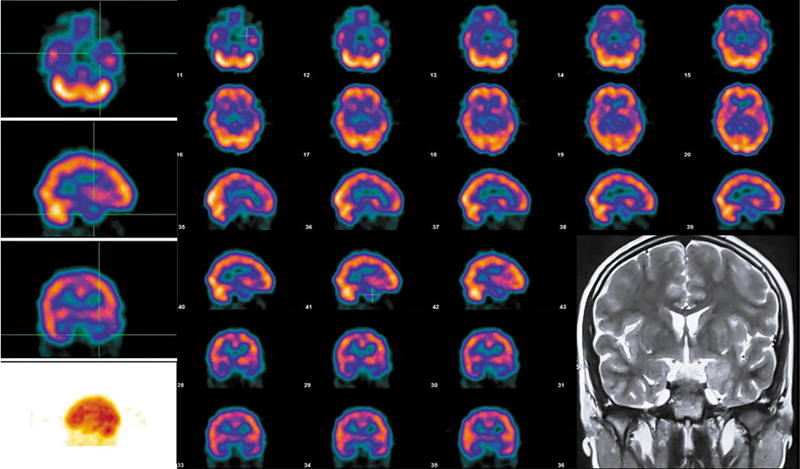
Interictal 99mTc-ethyl cysteinate dimer (ECD) brain perfusion single photon emission computed tomography (SPECT) in a 12-year-old male child with right-sided focal epilepsy of 8 years' duration, magnetic resonance imaging (MRI) coronal sections show left-sided mesial temporal sclerosis. SPECT images showing moderate to severe hypoperfusion involving left medial temporal cortex and mild hypoperfusion involving right medial temporal cortex.

In our study, 6/60 children had drug-resistant TLE in which 3/60 (5%) had mesial temporal lobe sclerosis. Additionally, the authors reviewed usage of resective surgery, selective amygdalohippocampectomy, gamma knife stereotactic radiosurgery, stereotactic laser thermoablation, neuromodulatory devices, and underutilization of surgical treatment in DRE, a scenario that was similarly observed in our study ( Fig. 2 ).

The research points out the difficulties in using structural imaging to identify epileptogenic lesions, as a substantial number of patients had no lateralization detectable on MRI even though they clearly were lateral on 99mTc-ECD. This aligns with a recently published review that identified a definitive cause of epilepsy in 33% of children with epilepsy, while the remaining two-thirds remained unidentified despite the use of advanced imaging and diagnostic techniques. The most common causes identified were structural abnormalities and genetic factors.

Current research is concerned with the role of 99mTc-ECD in revealing lateralization. Certain factors like epilepsy type, structural anomalies, or the timing of the imaging with respect to seizures may affect the diagnostic accuracy of 99mTc-ECD. On follow-up, 75% of patients achieved clinical control, whereas 25% did not. The mortality rate stood at 1.7%, with the unfortunate demise of one patient. In nearly all cases (98.3%), management determined that no alterations were required. According to the results, the majority of patients obtained positive clinical control outcomes without requiring any changes to their treatment plan. Consistent with previous studies on effective epilepsy treatment, this investigation found that 75% of patients attained clinical control.

Based on a recent review on epilepsy surgery by Rugg-Gunn et al, significant proportion of patients (30–40%) attained freedom from seizures from focal epilepsy, following epilepsy surgery. Surgical treatment of refractory TLE improves both seizure freedom and improved quality of life compared with optimal medical management. 18

The current study did not particularly analyze the results of surgery as only one patient with craniopharyngioma was advised for surgery but declined by his parents due to personal reasons. However, 45/60 (75%) patient in the current cohort were clinically controlled with AEDs, suggesting that a significant proportion of patients can achieve seizure cessation with the right treatment. In this study cohort, a small percentage (1/60 [1.7%] patient with craniopharyngioma) required management change.

Epilepsy-associated mortality imposes a significant burden on the public health of high-income countries. Important causes of death among people with epilepsy include injuries, status epilepticus, and sudden unexpected death in epilepsy, which may be preventable with access to high-quality specialty health care. A recent systematic review by Thurman et al on burden of premature mortality of epilepsy, reported considerable high standardized mortality ratio for epilepsy in children ranging from 6.4 to 7.5. This indicates seven times increased risk of death in children compared to the general population. 19 In our study a lower mortality rate (1.7%) could possibly be due to relatively short follow-up duration and a small sample size.

Functional neuroimaging with brain perfusion SPECT has an important role in (1) MRI negative nonlesional epilepsy, (2) discordance between electroclinical (EEG) and structural localization, (3) when there are multiple lesions, and (4) in preoperative setting. 20

In our study, all patients showed abnormal interictal brain perfusion SPECT. Moderate to severe hypoperfusion was seen in left temporal lobe in 39/60 (65%) patients, whereas 30/60 (50%) patients showed moderate to severe hypoperfusion in right temporal lobe.

Medial temporal lobe hypoperfusion was seen in majority of patients, while 26.67% patients had additional foci of hyperperfusion in left frontal lobe and 23.3% patients showed right frontal lobe defects. Unilateral hemisphere involvement was seen only in 60% patients and bilateral hemispheric involvement in 40% patients ( Table 6 ). SPECT findings were concordant with EEG in 16/60 (26.67%) of cases and concordant MRI, EEG, and 99mTc-ECD was seen in only 5/60 (8.3%) cases. Many other factors such as age-related cortical maturation, the inherent reduced uptake of 99mTc-ECD in the medial temporal lobe must be considered before interpreting ECD studies.

In tune with previous publication, our study highlights the utility of 99mTc-ECD SPECT alone in providing additional diagnostic insights, where other modalities were either unremarkable or discordant. This could significantly influence patient management and outcomes. Structural imaging with MRI provides insight regarding the structural abnormalities and when integrated with functional neuroimaging techniques, it significantly improves the precision of seizure onset zone (SOZ) localization. 21

Although interictal SPECT studies may add useful information to ictal studies, it cannot be recommended as a sole diagnostic procedure for focus detection. This was a major limitation of our study. The evolution of SISCOM (subtraction ictal SPECT coregistered to MRI), SISCOS (subtraction ictal SPECT coregistered to interictal brain SPECT), and PISCOM (PET interictal subtracted ictal SPECT coregistered with MRI) has further refined the interpretation of brain perfusion SPECT studies for more accurate and objective localization of SOZ. 21 Another limitation includes for patients with a seizure onset duration of less than 1 year ( *n* = 19, 37%) could not be accurately assessed for DRE compared to those with a duration greater than 1 year ( *n* = 41). Follow up of patients with less than 1 year ( *n* = 19, 37%) history could not be possible.

18F FDG-PET is more sensitive than interictal SPECT and has similar sensitivity to ictal SPECT for presurgical localization of epileptic foci in patients with noncontributory EEG and normal MR. FDG-PET also provides additional information on the functional status of the rest of the brain. 22

## Conclusion

The study aimed to evaluate brain perfusion patterns in children with DRE using 99mTc-ECD brain perfusion SPECT. Among the 60 patients assessed, the cohort displayed a heterogeneous range of clinical presentations, with right-sided and left-sided focal seizures being the most common.

The findings revealed significant variability in the lateralization of the epileptogenic focus across different diagnostic modalities—clinical evaluation, MRI, EEG, and 99mTc-ECD brain perfusion SPECT. Notably, 99mTc-ECD SPECT proved particularly valuable in cases where MRI and EEG were inconclusive, highlighting its importance in the comprehensive assessment of DRE. 99mTc-ECD SPECT stands out for providing additional diagnostic insights, can serve as complementary tool when other modalities are either unremarkable or discordant, thereby greatly influencing patient management and outcomes.
